# Differential prognostic values of the three *AKT* isoforms in acute myeloid leukemia

**DOI:** 10.1038/s41598-024-57578-x

**Published:** 2024-03-25

**Authors:** Eulalie Corre, Cécile Soum, Romain Pfeifer, Chloé Bessière, Sandra Dailhau, Catherine Marbœuf, Fabienne Meggetto, Christian Touriol, Christian Récher, Marina Bousquet, Stéphane Pyronnet

**Affiliations:** 1 Centre de Recherches en Cancérologie de Toulouse (CRCT), INSERM UMR-1037, CNRS UMR-5071, Université de Toulouse, Toulouse, France; 2 Service d’Hématologie, Centre Hospitalier Universitaire de Toulouse, Institut Universitaire du Cancer de Toulouse Oncopôle, Toulouse, France

**Keywords:** Haematological cancer, Tumour biomarkers

## Abstract

The PI3K-AKT-mTOR pathway lies at the confluence of signaling pathways in which various components are subjected to activating genetic alterations in acute myeloid leukemia (AML), thus contributing to oncogenesis. Three *AKT* isoforms exist in humans. However, whether one isoform predominates in AML remains unknown. This study reveals that *AKT3* behaves very distinctly than *AKT1* or *AKT2* in both normal myeloid differentiation and AML. During normal differentiation, *AKT3* is preferentially expressed in hematopoietic stem cells whilst *AKT1* becomes preferentially expressed as cells differentiate into granulocytes or monocytes. *AKT2* expression remains unchanged. In AML, *AKT3* expression varies widely among patient samples and is counterintuitively high in mature/monocytic leukemia. Furthermore, a low level of *AKT3* expression is strongly correlated to genetic alterations associated with a better outcome (*NPM1* mutations and *RUNX1*-*RUNX1T1* translocation), while a high level is correlated to alterations associated to a bad outcome (*RUNX1* mutations; and *SRSF2*, *U2AF1*, *SF3B1*, *ASXL1* and *BCOR* mutations occurring frequently in MDS and MPN). Consistently, a high *AKT3* expression level appears as a very strong predictor of poor survival. Curiously, although modestly varying among AML samples, a high *AKT1* expression shows in contrast as a strong predictor of a better patient outcome. These data suggest that *AKT3* and *AKT1* expressions have strong, yet opposite, prognostic values.

## Introduction

Acute myeloid leukemia (AML) is a heterogeneous disease. It is characterized by a plethora of genetic alterations including various chromosomal translocations and/or a wide variety of mutations, whose nature allows a classification of patients according to their chances of survival. One classification that has been accepted for several years is that given by the European LeukemiaNet in 2017 (ELN-2017^1^) and updated in 2022 (ELN-2022^2^). Depending on the nature of the cytogenetic abnormalities and mutations detected at diagnosis, this classification divides patients into three groups: the *adverse* group with a risk of poor survival, the *favorable* group with an expected better prognosis, and an *intermediate* group. However, even if this scoring remains a good assessment, the survival of patients in each of these groups can be heterogeneous. The detection of genetic abnormalities at diagnosis can also assist clinicians in recommending targeted therapies, typically prescribed as a second-line treatment. Indeed, the majority of AML patients are first treated with intensive chemotherapy to harness the lethal acute phase. Although some patients are refractory, many go into remission after this induction chemotherapy. To reinforce the treatments and to delay the relapses, patients can then benefit from a milder protocol known as consolidation chemotherapy, and/or benefit from a targeted therapy. There are therapies specifically targeting genetic alterations, such as inhibitors of the mutated FLT3 kinase, inhibitors of the mutated IDH1 and IDH2 enzymes, or molecules targeting DNA methylation, as frequent mutations affect various genes (e.g. *DNMT3A*, *TET2*) whose protein products are involved in the regulation of DNA methylation. Targeted therapies include also BCL2 inhibitors, prescribed mainly to unfit patients who are not eligible for intensive chemotherapy. A wide range of molecules targeting less specific features has also been tested in pre-clinical settings as potential therapeutics against AML. These include molecules impinging upon major signaling pathways that are activated in leukemic cells but also in many other cancers. This is for instance the case for compounds inhibiting the AKT protein kinase.

AKT is a serine/threonine kinase playing major roles in intracellular signaling pathways. It is one key actor in the PI3K-AKT-mTOR pathway which senses environmental signals such as growth factors and nutrients availability, and converts these cues into survival, growth and/or proliferation capabilities. Of note, the PI3K-AKT-mTOR pathway controls the rate of protein synthesis and consequent cell growth^3^, a pre-requisite for entering the cell cycle. Given these critical properties, AKT has attracted much attention from the medico-scientific community, particularly in the cancer field, and AKT inhibitors have been designed for research purposes and for clinical perspectives^4^. Although *AKT* itself is rarely mutated in AML, the kinase lies at the confluence of several signaling pathways in which various components can themselves be activated by gene mutations (e.g. *FLT3*, *KIT*, *NRAS*, *KRAS*, *PIK3CB*…). However, the use of AKT inhibitors in pre-clinical settings has been disappointing, with an overall non-convincing benefit-to-risk ratio. After all, AKT also plays important physiological roles in healthy cells. Targeting AKT can therefore provoke significant adverse effects which limit the applicable doses. Yet, there are three distinct *AKT* genes in human, encoding three different proteins: AKT1, AKT2 and AKT3. These three AKT proteins show tissue-specific distributions^5^ and recent studies indicate that each may exhibit different affinities for various partners and may therefore touch more specifically one pathway than another^6^. Indeed, although the three human AKTs possess a relatively well conserved overall amino-acid sequence, they harbor some sequence divergences and distinct intracellular localizations. Both of these features might perhaps explain the fact that they can interact with and phosphorylate different partners. However, although some AKT1 versus AKT2 substrate specificities have been identified, much less is known about AKT3 specific targets^6^. In a more recent report, while AKT1 was described as preferentially located in close proximity of the plasma membrane, AKT2 appeared more cytosolic and AKT3 seemed anchored to the nuclear envelope, facing the cytosol^7^. In the same report, AKT3 has been suspected to be constitutively activated in a series of established cancer cells (no leukemic cells were included in this study), although the mechanism responsible for such a permanent activation remains unknown. This observation reinforced an earlier report saying that AKT3 is also constitutively activated in a glioma cell line^8^. Unfortunately, none of the AKT inhibitors available to date exhibit a sufficiently strong isoform specificity to selectively target AKT3^6^. Thus, the development of inhibitors specific to each AKT isoform could be of clinical interest.

In this study, we examined the expression of the three *AKT* isoforms in a thousand of normal hematopoiesis and AML samples emanating from cohorts established in three different countries (France, USA, Canada). All the data clearly converge toward *AKT3* as being the bad in AML.

## Results

### Expression of the three AKTs in normal myeloid differentiation

To generate a picture of how expression of each of the three *AKT* genes evolves in the course of differentiation of the myeloid lineages, analyses of healthy samples from the Bloodspot^9^ and Leucegene public datasets were performed.

**Figure 1:**
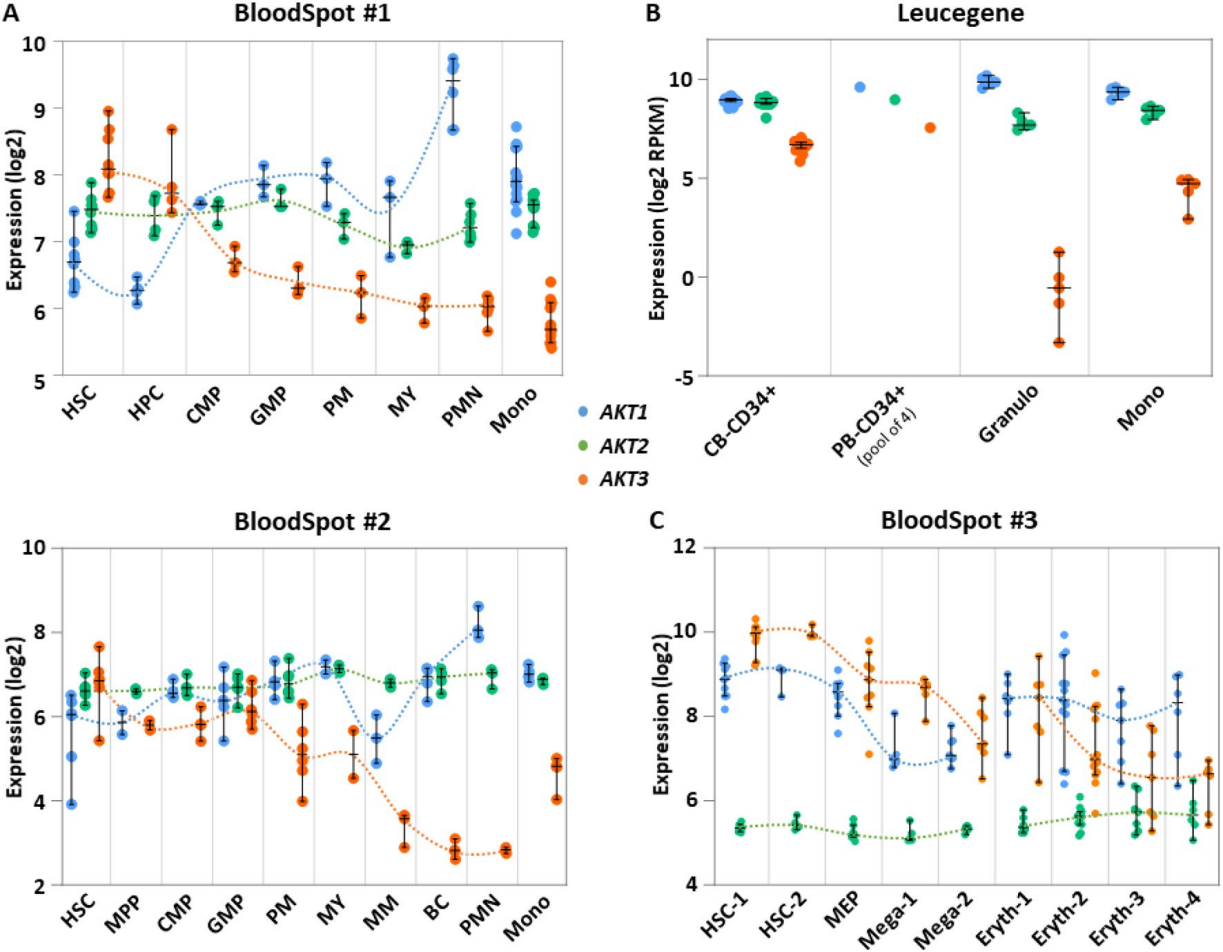
Expression of each *AKT* transcript in normal hematopoiesis. (**A**) Each dot in the curves shows micro-array normalized expression (log2) of each *AKT* transcript in cells sorted from individual healthy bone marrow donors of the BloodSpot #1 (top) or #2 (bottom) series of samples as described in the Method section. X axes: HSC = hematopoietic stem cell; HPC = hematopoietic progenitor cell; MPP = multipotent progenitor; CMP = common myeloid progenitor; GMP = granulocyte monocyte progenitor; PM = promyelocyte; MY = myelocyte; MM = metamyelocyte; BC = band cell; PMN = polymorphonuclear cell; Mono = monocyte. Array probes: 207163_s_at (AKT1), 225471_s_at (AKT2), 212609_s_at (AKT3) (top); 207163_s_at (AKT1), 225471_s_at (AKT2), 212607_at (AKT3) (bottom). Other probes gave similar tendencies. (**B**) Each dot shows RNA-seq normalized expression (log2 RPKM) of each *AKT* transcript in cells sorted from healthy donors of the Leucegene series as described in the Method section. X axis: CB CD34 +  = cord blood CD34 + cells of individual donors; PB CD34 +  = peripheral blood CD34 + cells pooled from 4 donors; Granulo = granulocytes; Mono = monocytes. (**C**) Each dot in the curves shows micro-array normalized expression of each *AKT* transcript in cells sorted from individual healthy bone marrow donors of the BloodSpot #3 series of samples as described in the Method section. X axis: HSC-1 = HSC CD133 + CD34dim; HSC-2 = HSC CD38- CD34 + ; MEP = megakaryocyte/erythrocyte progenitor; Mega-1 = CFU megakaryocyte; Mega-2 = megakaryocyte; Eryth-1 = eryth.CD34-CD71 + GlyA-; Eryth-2 = eryth.CD34-CD71 + GlyA + ; Eryth-3 = eryth.CD34-CD71loGlyA + ; Eryth-4 = eryth.CD34-CD71-GlyA + . Array probes: 207163_s_at (AKT1), 211453_s_at (AKT2), 212607_at (AKT3). Bars represent medians + /- 95% CI (confidence interval).

We first looked at two independent microarray datasets from the BloodSpot repository (datasets #1 and #2), each containing peripheral blood or bone marrow cells sorted by cytometry with the help of specific markers distinguishing different steps of myeloid differentiation, staging from hematopoietic stem cells (HSCs) to granulocytes or monocytes. In both sets, the data revealed that *AKT1* expression shows a bi-phasic but significant increase along granulo or monocytic differentiation, although at the end AKT1 expression appears higher in granulocytes than in monocytes. *AKT2* expression remains constant. The expression of *AKT3* in HSCs appears in both sets higher than those of *AKT1* and *AKT2* then decreases gradually but strongly as cells progress toward terminal granulocytes or monocytes, although the decrease is less marked in monocytes of the dataset #2 (Fig. 1A, top and bottom).

To verify these observations in samples from healthy donors where gene expression was measured by another technique, we looked at bulk RNA-seq data of the Leucegene project. This dataset contains sorted CD34+ HSC/progenitor samples from either cord of peripheral blood and granulocytes and monocytes from peripheral blood. As compared to the observations made in BloodSpot microarray datasets #1 and #2, a similar observation was made, with notably a significant lower expression of *AKT3* in granulocytes and monocytes (although less marked in monocytes) than in CD34+ HSC/progenitors (Fig. 1B).

Expression of each the three *AKT*s was then monitored in samples of a third BloodSpot dataset (#3) which contains, among others, normal hematopoietic samples that were sorted with the help of antibodies specific to various stages of differentiation including HSCs, megakaryocytic/erythroid progenitors and intermediary and terminal megakaryocytes and erythrocytes. *AKT1* expression goes down as cells differentiate into megakaryocytes, but does not change during erythroid differentiation. *AKT2* expression remains always constant, while that of *AKT3* diminishes gradually along differentiation into both terminal megakaryocytes and terminal erythrocytes (Fig. 1C).

Taken together, analyses of these four independent data sets converge toward a similar observation: *AKT3* expression is elevated in HSCs but goes down as they differentiate into either one of the four branches (granulocytic, monocytic, megakaryocytic or erythroid) of myeloid differentiation.

### Expression of the three AKTs in AML samples

**Figure 2:**
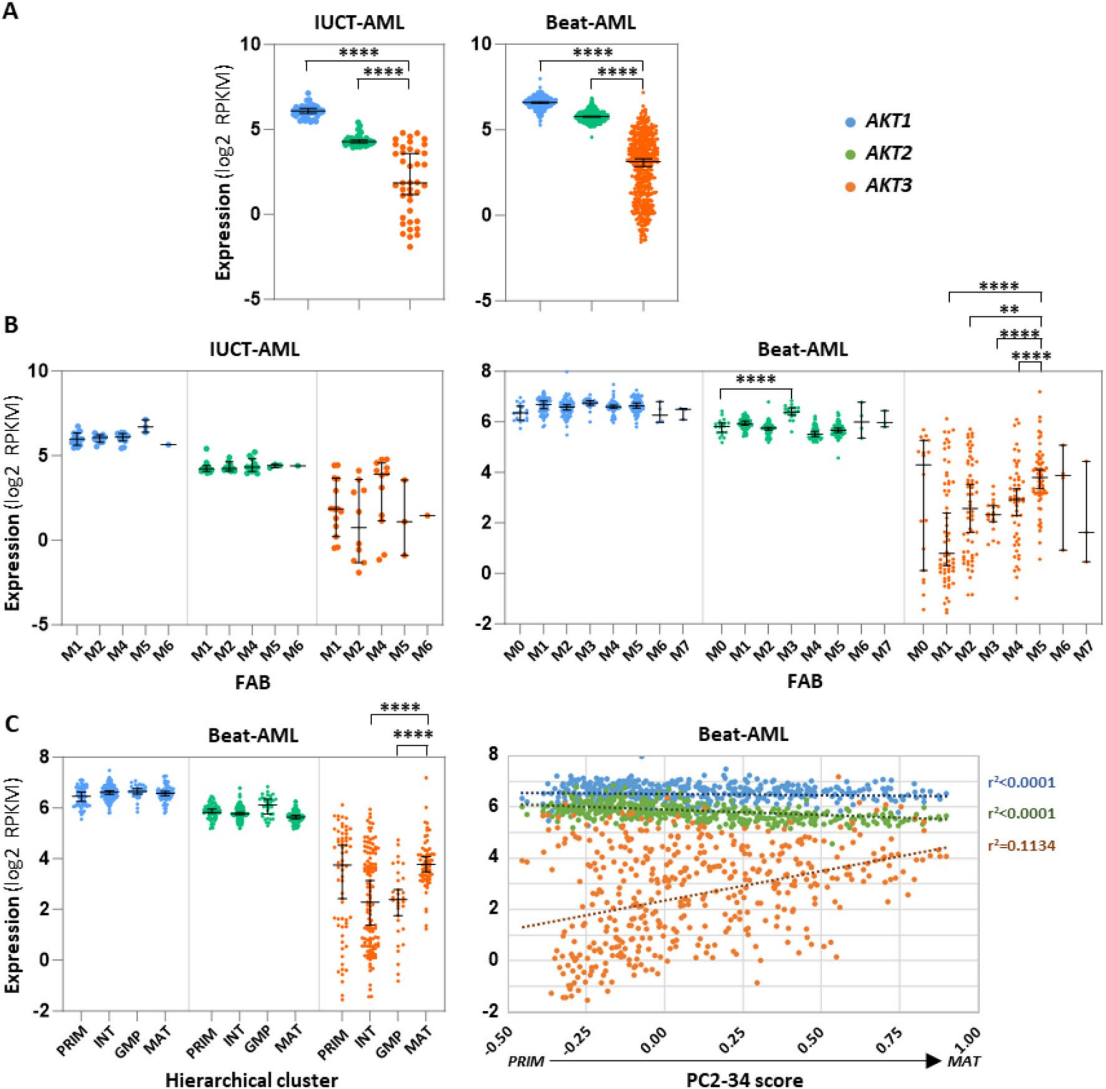
Expression of each *AKT* transcript in AML samples. (**A**) Each dot shows RNA-seq normalized expression (log2 RPKM) of each *AKT* in samples of the IUCT-AML (left, n = 40) or the Beat-AML (right, n = 445) datasets. (**B**) Each dot shows expression of each *AKT* in AML FAB categories of the IUCT-AML (left, n = 40) or of the Beat-AML (right, n = 445) cohort. X axes: M0 = undifferentiated; M1 = myeloblastic without maturation; M2 = myeloblastic with maturation; M3 = promyelocytic; M4 = myelomonocytic; M5 = monocytic; M6 = erythroleukemia; M7 = megakaryoblastic. (**C**) Each dot shows expression of each *AKT* in hierarchical clustering categories (left, n = 281) or as a function of the PC3-34 score (right, n = 445) of the Beat-AML series of samples. X axes: PRIM = *primitive*; INT = *intermediary*; GMP = *granulo-monocyte progenitor*; MAT = *mature*. Bars represent medians with 95% CI. Trend curves are shown as dotted lines + coefficients of linear regression (r^2^).

*Global expression* We first analyzed a bulk ribo-zero RNA-seq we have performed earlier on the blasts of 40 AML patients, a cohort hereafter named IUCT-AML, and in which all samples are with normal cytogenetics (CN-AML)^10^. The results indicate that expression of *AKT3* in blasts is globally weaker than those of *AKT1* and *AKT2*. However, the range of *AKT3* expression shows a much wider spectrum among samples, with a roughly ~ 120-fold difference between the sample expressing the most and the one expressing the less *AKT3*. In the same samples, the variations in *AKT1* and *AKT2* expressions are very much less marked with an only ~ threefold amplitude for each isoform (Fig. 2A, left). To gain in significance, the expression of *AKT* was verified in 445 AML diagnosis samples from the Beat-AML public data set (including samples with either normal or abnormal cytogenetics)^11^ where gene expression was measured by poly(A) RNA-seq. Similar observations were made. Clearly, although globally weaker, *AKT3* expression among AML samples is much more variable than those of *AKT1* and *AKT2* (Fig. 2A, right).

*FAB.* We then asked whether expression of each *AKT* in blasts shows a pattern similar to the one seen during normal differentiation, with notably a decreasing expression of *AKT3* as cells progress toward differentiation. To obtain a first indication, we looked in AML samples categorized according to the French-American-British (FAB) classification. Such FAB classification is based on blast morphological aspects and groups AML samples into seven categories (M0-M7), from the less (M0) to the most (M5: monoblastic leukemia; M6: erythroleukemia; M7: megakaryocytic leukemia) differentiated stages. In our IUCT-AML series, which is devoid of M0, M3 and M7 samples, the M4 group contains samples with the highest level of *AKT3* expression, although such a visible tendency is not statistically significant. No conclusion can be drawn from the M5 and M6 categories as they contain only 3 and 1 samples, respectively (Fig. 2B, left). However, among the bigger number of Beat-AML diagnosis samples for which the FAB is known (n = 275) and containing many samples in all FAB categories, a significantly higher expression of *AKT3* in the M5 category appears, with a noticeable progressive increase in *AKT3* expression from M1 to M5 groups. The M3 group, also called “acute promyelocytic leukemia”, and which represents a separate entity characterized by the *PML*-*RARA* chromosomal translocation, shows a homogenous pattern of *AKT3* expression, and a significant ~ twofold increase in *AKT2* expression. Another intriguing observation is that Beat-AML samples of the M0 and M1 (and to a lesser extent of the M2 and M4) categories seem to cluster into two *AKT3*^high^ and *AKT3*^low^ subgroups. No conclusion can be drawn from the M6 and M7 categories as they contain only 6 and 3 samples, respectively (Fig. 2B, right). Thus, *AKT3* expression in AML samples is apparently following a counterintuitive pattern with the M4/M5 myelomonocytic/monocytic leukemia samples exhibiting the highest levels of AKT3 expression, as opposed to what happens in normal differentiation.

*Hierarchical clustering and PC2-34 scoring.* As the FAB classification is based only on morphological characteristics, it was important to explore the expression level of each *AKT* by another approach, especially for the counterintuitive high level of *AKT3* expression seen in more mature M4/M5 samples. To do so, we performed new RNA-seq analyses in light of a recent publication which, based on gene expression deconvolution on bulk AML transcriptomes (from TCGA, Beat-AML and Leucegene projects) using single-cell reference profiles of distinct AML stem, progenitor and mature cell types, indicates that AML samples can be categorized into four hierarchical clusters of different degrees of maturation: *primitive* (shallow hierarchy, LSPC-enriched), *GMP* (dominated by GMP-like blasts), *mature* (steep hierarchy, enriched for mature Mono-like and cDC-like blasts) and *intermediate* (balanced distribution)^12^. Based on the combined and weighted expression of 34 genes, the authors also defined a score (called PC2-34) which can rank AML samples along the *primitive*-to-*mature* branch. First, thanks to the analysis performed earlier with 281 AML samples of the Beat-AML project^12^, we verified how *AKT3* expression distributes in the four hierarchical clusters. As expected from the FAB data presented above, all Beat-AML samples belonging to the *mature* cluster express a higher level of *AKT3* as compared to samples of the less mature *GMP* cluster. In the *primitive* and *intermediary* clusters, AML samples distribute into two sub-groups with different levels of *AKT3* expression (Fig. 2C, left). We then looked at the expression of each *AKT* as a function of the PC2-34 score in the 445 AML samples collected at diagnosis. The data show that as the score increases, i.e. as samples become increasingly *mature*, the level of *AKT3* expression augments globally except for a small subgroup of *primitive* samples where AKT3 expression is high. Expressions of *AKT1* and *AKT2* remain relatively constant (Fig. 2C, right).

Taken together, these data suggest that the regulatory mechanisms targeting expression of each *AKT* in leukemic blasts are distinct than those involved in normal myeloid differentiation, with notably an unexpected high level of *AKT3* expression in mature/monocytic leukemia but a lower level in many immature samples.

### Expression of the three AKTs and ELN-2017

**Figure 3:**
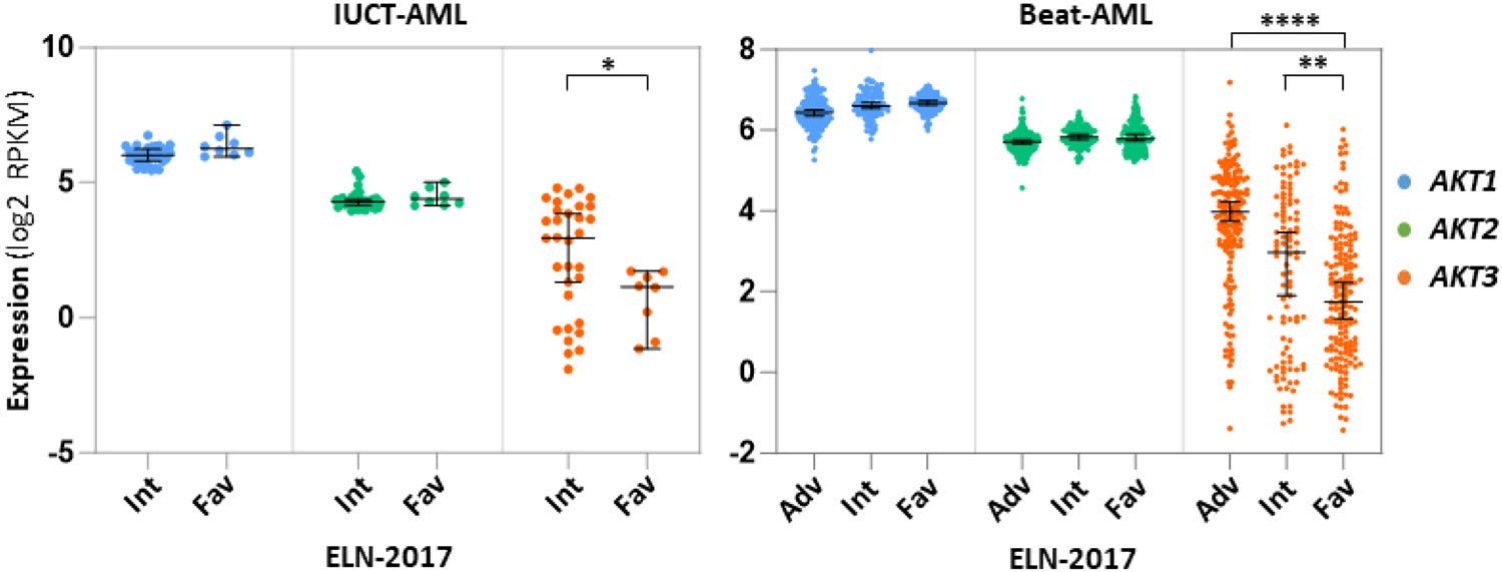
Expression of each *AKT* transcript as a function of ELN-2017 score. Each dot shows expression (log2 RPKM) of each *AKT* as a function of ELN-2017 score in samples of the IUCT-AML (left, n = 40) or of the Beat-AML (right, n = 429) cohort. X axes: Adv = *adverse*; Int = *intermediary*; Fav = *favorable*. Bars represent medians with 95% CI.

As *AKT3* expression is highly variable among AML samples, we thought it could be significantly correlated to some clinical parameters. We first looked at the expressions of each *AKT* as a function of the ELN-2017 score which distributes the patients into three groups of differently predicted outcomes: *adverse*, *intermediate* or *favorable*. Here again, *AKT3* is clearly distinguishable from the other two *AKT*s. Unfortunately, in our IUCT-AML cohort encompassing 40 AML samples, no sample is categorized as *adverse*, but 8 are in the *favorable* group and 32 are considered as *intermediate*. Despite the lack of an ELN-2017 *adverse* group, a strong difference in *AKT3* expression is visible between the *intermediate* and the *favorable* groups, a low expression of *AKT3* being a clear mark of the *favorable* entity (Fig. 3, left). We then looked at the Beat-AML diagnosis samples for which the ELN-2017 is known (n = 429) and where there is a copious number of samples in each of the three ELN-2017 categories. Consistently with our cohort, samples of the *favorable* group are those expressing very significantly the less *AKT3* as compared to samples of the *adverse* and *intermediate* groups (Fig. 3, right). In both cohorts, a slightly higher expression of *AKT1* appears in contrast in the *favorable* group, although the differences are much less pronounced, likely because the expressions of both *AKT1* and *AKT2* are much less variable among samples than that of *AKT3* (Fig. 3).

### Expression of the three AKTs and genetic status

**Figure 4:**
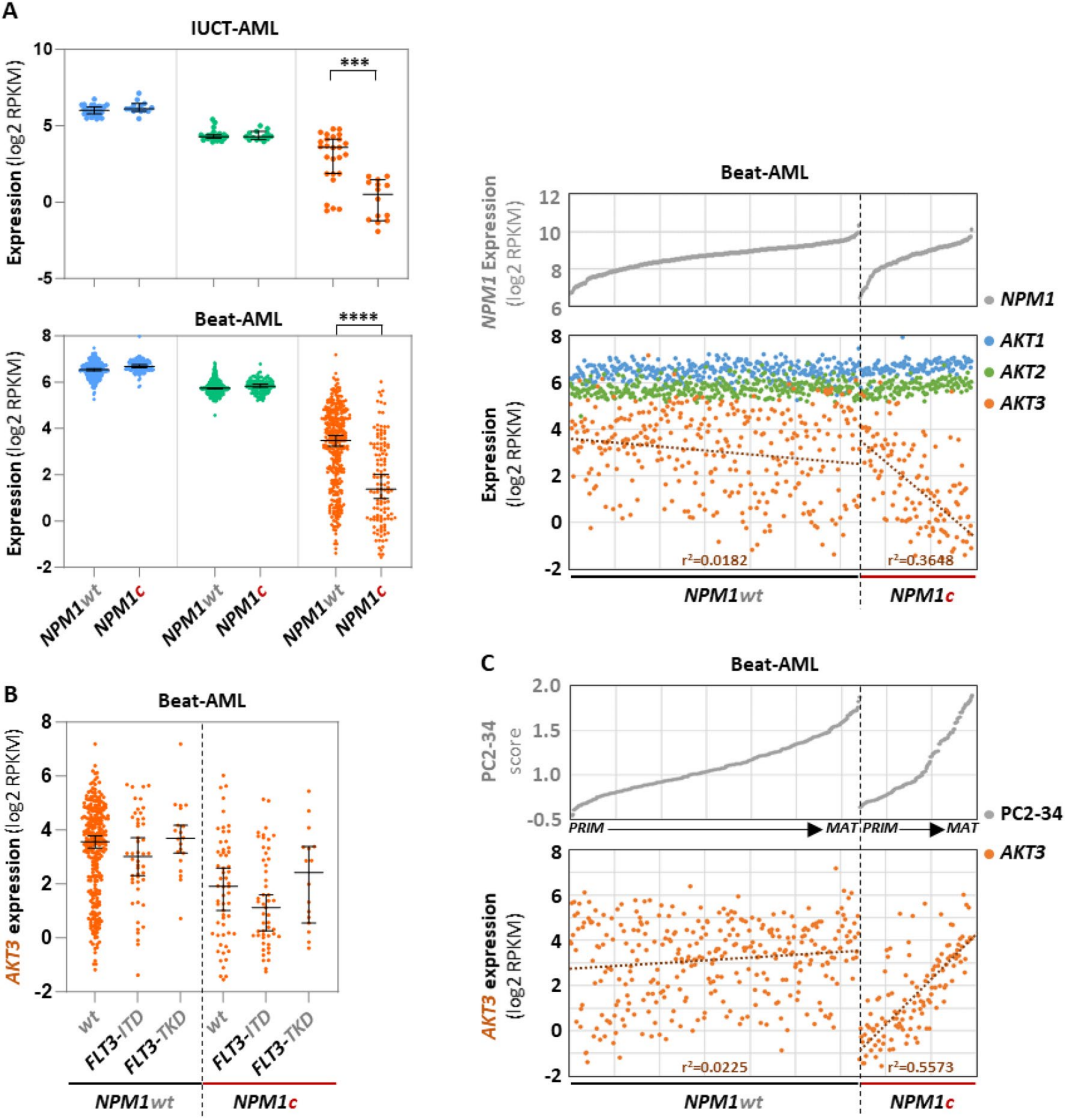
Expression of each *AKT* transcript as a function of *NPM1* and/or *FLT3* mutational status. (**A**) Each dot shows expression (log2 RPKM) of each *AKT* as a function of *NPM1* mutational status in samples of the IUCT-AML (top left, *NPM1c* = 14/40) or of the Beat-AML (bottom left, *NPM1c* = 125/445). Expression of each *AKT* in the Beat-AML cohort (n = 445) as a function of *NPM1* expression in *NPM1wt* (n = 320) or *NPM1c* (n = 125) genotype, samples being ranked for each genotype in ascending order of global *NPM1* expression (right). (**B**) Each dot shows expression of *AKT3* in 445 samples of the Beat-AML cohort as a function of *FLT3-ITD* alone (n = 48), *FLT3-TKD* alone (n = 20), *NPM1c* alone (n = 58), *NPM1c* + *FLT3-ITD* (n = 51) or *NPM1c* + *FLT3-TKD* (n = 16) genotype. “*wt*” means no *FLT3* or *NPM1* mutation. (**C**) Expression of *AKT3* in 445 samples of the Beat-AML cohort as a function of the PC2-34 score in *NPM1wt* (n = 320) or *NPM1c* (n = 125) genotype, samples being ranked for each genotype in ascending order of PC2-34 score. PRIM = *primitive*; MAT = *mature*. Bars represent medians with 95% CI. Trend curves are shown as dotted lines + coefficients of linear regression (r^2^).

*NPM1* mutations. Since the ELN-2017 score is based on the nature of the genetic alterations found in AML samples and because *AKT3* expression is significantly higher in the *adverse* group than in the *favorable* and *intermediate* groups, it was probable that some of these genetic abnormalities could be either correlated or anti-correlated to the level of *AKT3* expression. We therefore looked at the expressions of the three *AKT* isoforms as a function of the most frequent mutations in our IUCT-AML cohort. *NPM1* is the most frequently mutated gene in AML (approximately 30% of patients). Mutations in *NPM1* create a sequence frameshift converting the nuclear localization signal located in the carboxyl-terminus of the protein into a nuclear export signal. As a consequence, the mutant proteins are localized in the cytosol (and are called NPM1c), while the wild-type protein (NPM1wt) shuttles between the nucleus and the cytoplasm. Interestingly, a lower level of *AKT3* expression is clearly visible in the 14 *NPM1c* AML samples as compared to the 26 *NPM1wt* samples (Fig. 4A, top left).

To confirm this observation in a bigger series of samples, we repeated this analysis in the Beat-AML cohort which contains 125 *NPM1c* samples out of 445 AML diagnosis samples for which both the *NPM1* mutational status and the RNA-seq data are available. Consistently with our modest cohort, a strong correlation exists here between *NPM1c* and a low expression of *AKT3* (Fig. 4A, bottom left). The expression level each *AKT* isoform was then plotted as a function of *NPM1* expression in either *NPM1wt* or *NPM1c* samples. In *NPM1wt* samples, the expression of *AKT3* seems modestly inversely correlated to that of *NPM1* (r^2^ = 0.0182), while no correlation is detected with *AKT1* or *AKT2*. In *NPM1c* samples such anti-correlation between *AKT3* and *NPM1* expressions is much more pronounced (r^2^ = 0.3648) (Fig. 4A, right).

A gene whose mutations are frequently concomitant to those of *NPM1* is *FLT3*. *FLT3* can carry two types of mutations: internal tandem repeats (*FLT3-ITD*) and point mutations in the catalytic tyrosine kinase domain (*FLT3-TKD*). Whether or not these mutations coexist with that of *NPM1* is a parameter affecting patient outcome, especially the combination *NPM1c* + *FLT3-ITD* which is of *intermediary* prognosis, while *NPM1c* alone is of *favorable* prognosis. We have therefore checked whether the different combinations between *NPM1c* and/or *FLT3-ITD* or *FLT3-TKD* groups of patient samples can be distinguishable by different levels of *AKT3* expression. The results show no significant impact of any of the two types of *FLT3* mutations in *NPM1wt* as well as *NMP1c* backgrounds, (Fig. 4B). Thus, *NPM1c* remains the major event associated with a low level of *AKT3* expression in AML.

It has been often suggested that the *NPM1c* mutations in AML is characterized by *CD34* negativity and monocytic differentiation. Therefore, to verify whether or not the association between *NPM1c* and low *AKT3* expression is more related to the maturation status than the presence of *NPM1c*, *AKT3* expression levels were plotted as a function of the PC2-34 score in either *NPM1wt* or in *NPM1c* samples. While in *NPM1wt* samples a relative flat distribution (r^2^ = 0.0225) is observed, in *NPM1c* samples the level of *AKT3* expression is nicely correlated to the PC2-34 score (r^2^ = 0.5573) (Fig. 4C). This indicates that even if *AKT3* expression is globally low in the *NPM1c* AML entity, it strongly goes up as *NPM1c* samples loose in immaturity markers.

All together, these data clearly reveal two strong yet independent features associated with different levels of *AKT3* expression in AML: (i) many of the samples expressing the less *AKT3* are those carrying a mutated *NPM1* gene and, (ii) the low level of *AKT3* expression increases in *NPM1c* blasts as cells progress toward a mature phenotype (as opposed to what happens in normal hematopoietic differentiation).

**Figure 5:**
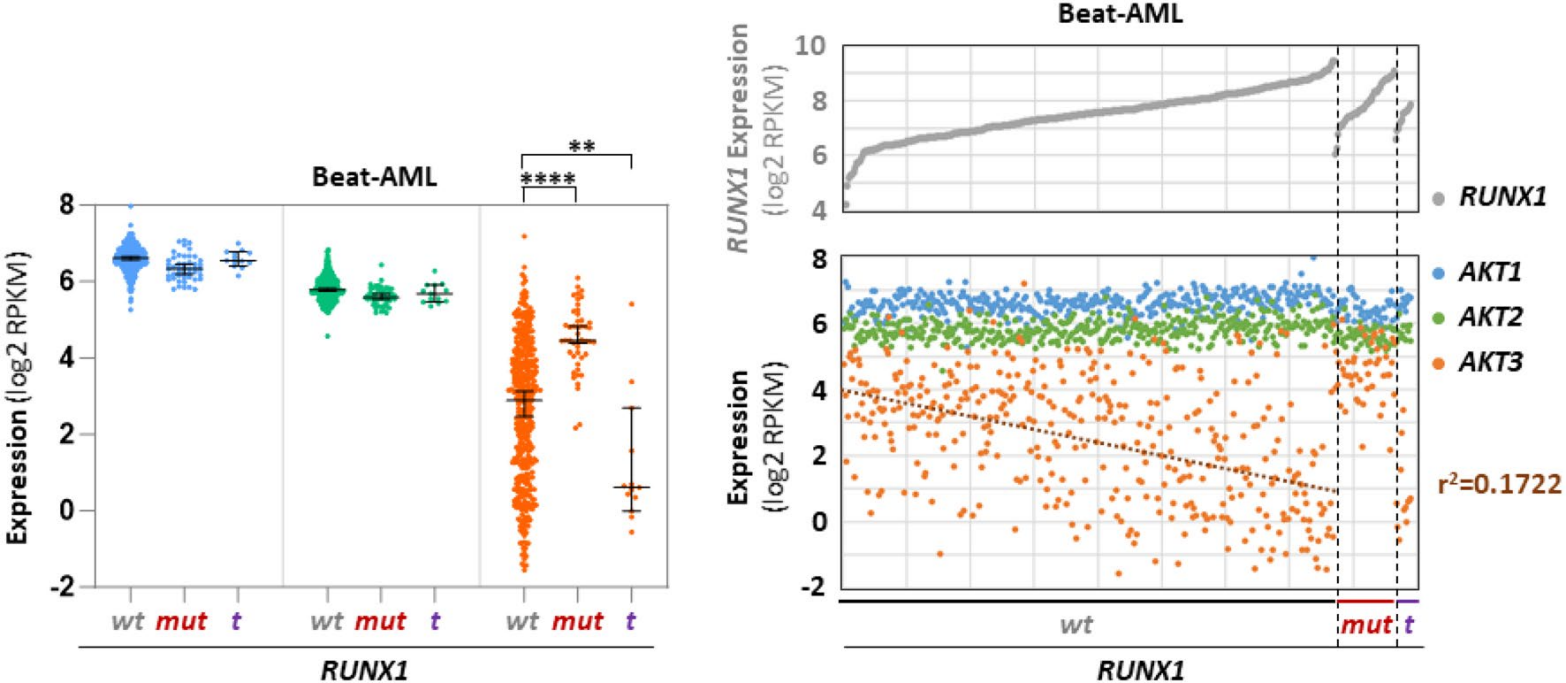
Expression of each *AKT* transcript as a function of *RUNX1* genetic status. Each dot shows expression (log2 RPKM) of each *AKT* as a function of *RUNX1* mutation (*mut*, n = 47) or *RUNX1-RUNX1T1* translocation (*t*, n = 13) in the 445 samples of the Beat-AML cohort (“*wt*” (n = 385) means no *RUNX1* mutation or translocation) (left). Expression of each *AKT* in the 445 samples of the Beat-AML cohort as a function of *RUNX1* expression in *RUNX1-wt* (*wt*, n = 385), *RUNX1-mut* (*mut*, n = 47) or *RUNX1-translocated* (*t*, n = 13) genotype, samples being ranked for each genotype in ascending order of global *RUNX1* expression (right). Bars represent medians with 95% CI. Trend curve is shown as dotted line + coefficient of linear regression (r^2^).

*RUNX1* mutations & *RUNX1*-*RUNX1T1* translocation. No significant correlation between expression of each of the three *AKT*s and any other mutation could be detected in our IUCT-AML cohort. However, this lack of correlation might be explained by the fact that many mutations are much less frequent than the mutations of *NPM1*, and therefore do not permit statistical analyses within a series containing 40 samples. However, correlations between the expression level of *AKT3* and the Runt-related transcription factor 1 (*RUNX1*) genetic alterations emerged in the 445 diagnosis samples of the Beat-AML cohort. Indeed, *AKT3* expression appears very significantly higher in the 47 samples harboring mutations in *RUNX1*, and in contrast significantly lower in the 13 samples harboring the t(8;21) (*RUNX1*-*RUNX1T1*) chromosomal translocation (Fig. 5, left). We then plotted le level of expression of the three AKT isoforms as a function of *RUNX1* expression in either *RUNX1wt*, *RUNX1mut* or *RUNX1-RUNX1T1* samples. In *RUNX1wt* samples, the expression of *AKT3* appears somehow inversely correlated to global expression of *RUNX1* (r^2^ = 0.1722), while no correlation is detected with *AKT1* or *AKT2*. In *RUNX1mut* and *RUNX1-RUNX1T1* samples no correlation between any of the three *AKT*s and global expression of *RUNX1* is visible (Fig. 5, right).

These data show a strong upregulation of *AKT3* in AML samples carrying a mutated *RUNX1* gene, but in contrast a downregulation in samples harboring the *RUNX1-RUNX1T1* translocation. Furthermore, the level of *AKT3* expression appears inversely proportional to that of *RUNX1* in *RUNX1wt* samples, a tendency no longer visible in *RUNX1* genetic variant samples.

**Figure 6:**
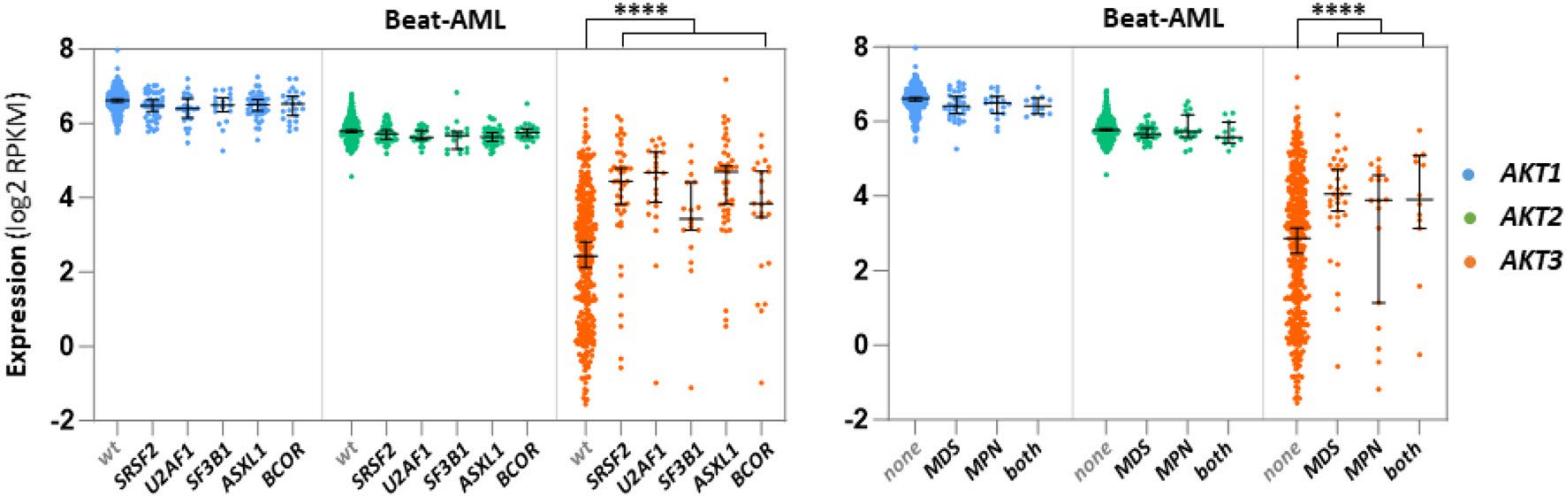
Expression of each *AKT* transcript as a function of *SRF2*, *U2AF1*, *SF3B1*, *ASXL1* or *BCOR* mutations and of prior MDS or prior MPN hematological disorders. Each dot shows expression (log2 RPKM) of each *AKT* as a function of *SRSF2* (n = 46)*, U2AF1* (n = 24)*, SF3B1* (n = 17)*, ASXL1* (n = 43) *or BCOR* (n = 23) mutations in 445 samples of the Beat-AML cohort (“*wt*” means no mutation in any of these 5 genes) (left), or in 445 samples but classified according to whether or not patients developed a MDS (n = 32), a MPN (n = 18) or a combination of both (n = 12) before the onset of leukemia (right). Bars represent medians with 95% CI. Student t tests were here calculated after having grouped mutant (left) or MDS + MPN + both (right) samples.

*SRSF2, U2AF1, SRSF3, ASXL1 and BCOR mutations, and relations with MDS and MPN.* In the 445 diagnosis samples of the Beat-AML cohort, a correlation between high levels of *AKT3* expression and mutations of *SRSF2* (n = 46), *U2AF1* (n = 24)*, SRSF3* (n = 17)*, ASXL1* (n = 43) or *BCOR* (n = 23) is also observed (Fig. 6, left). Mutations in these genes are very frequent in myelodysplastic syndromes (MDSs) and myeloproliferative neoplasms (MPNs). MDS and MPN are two hematological disorders at risk for the onset of AML, and mutations in the related genes are categorized in the ELN-2017 *adverse* group. It was therefore of interest to verify the level of expression of each *AKT* in AML samples that developed after a MDS or a MPN. Such analyses were performed in the Beat-AML cohort. The data obtained reveal that among the 445 samples collected at diagnosis, those from patients who developed a prior MDS or a prior MPN exhibit a higher level of *AKT3* expression (although not significant for the “prior MPN” category likely due to an insufficient number of samples), but not of *AKT1* or *AKT2* (Fig. 6, right).

These data show an upregulation of *AKT3* expression in AML samples carrying mutations in *SRSF2, U2AF1, SRSF3, ASXL1* or *BCOR*, and consistently in samples of patients who developed a leukemia after a MDS or a MPN.

### Expression of the three AKTs and overall survival

**Figure 7:**
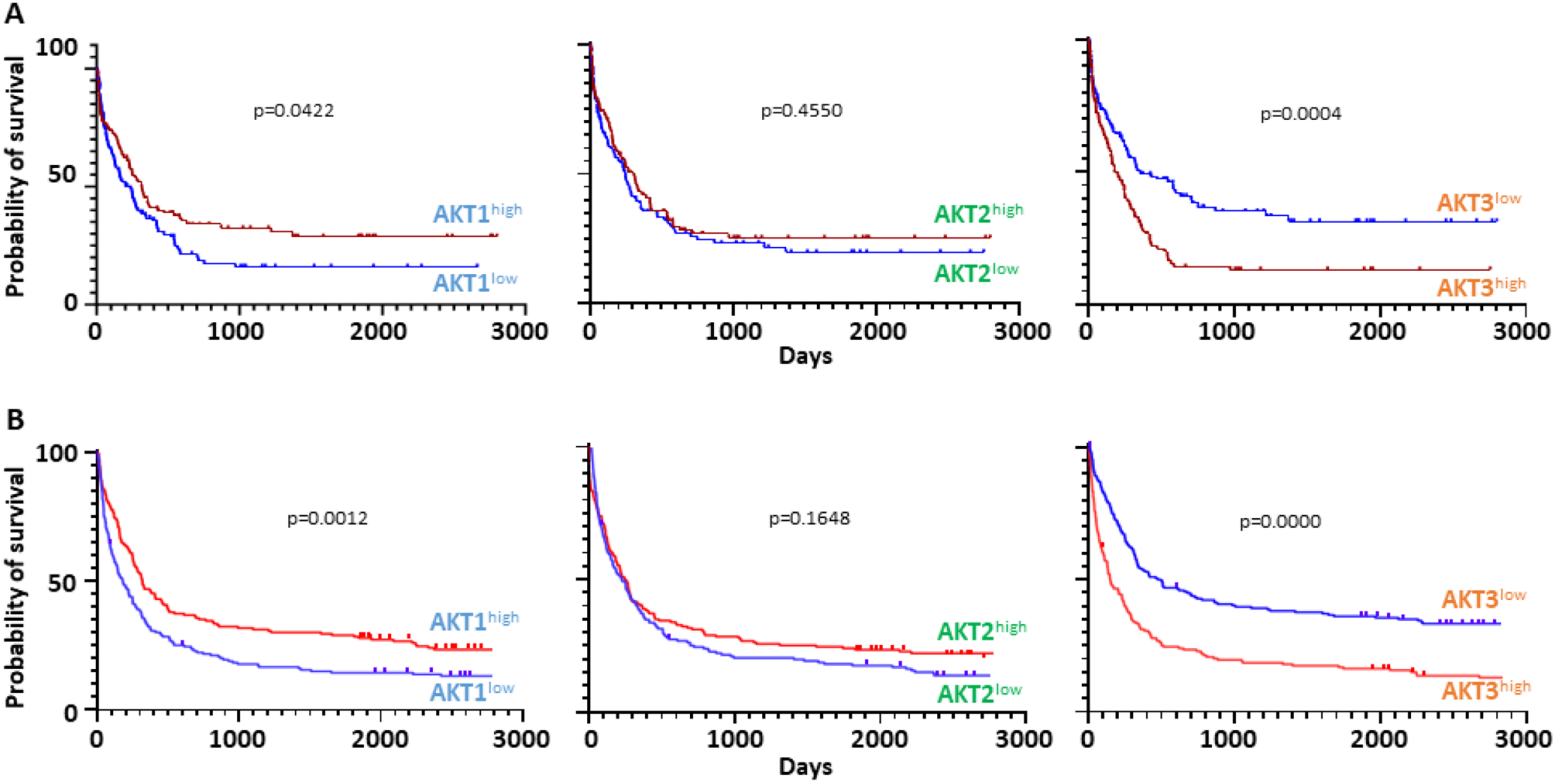
Kaplan–Meier curves showing the overall survival of AML patients as a function of expression each *AKT* transcript. (**A**) Data obtained from the Beat-AML cohort (n = 239). (**B**) Data obtained from the Leucegene cohort (n = 373).

All the aforementioned observations argued in favor of a bad prognostic value for *AKT3* expression. Indeed, samples of the ELN-2017 group express a higher amount of *AKT3*. Also, the level of *AKT3* expression is lower in leukemic blasts harboring *NPM1* mutations or the *RUNX1*-*RUNX1T1* translocation which are both of better prognosis, but higher in samples harboring *RUNX1, SRSF2, U2AF1, SF3B1, ASXL1, or BCOR* mutations which are of bad prognosis. To assess the prognostic impact of each *AKT*, the patient overall survivals were first evaluated by Kaplan–Meier curves using the Beat-AML dataset. To avoid potential bias in data interpretation, we removed here the samples that were not collected at diagnosis but later after the first line of therapy, those presenting with no AML but with an MDS or a MPN, and the samples from patients who could benefit from a transplantation (either bone marrow or cord blood cells engraftment). The Kaplan–Meier curves were finally plotted for each *AKT* with such a homogeneous set of n = 239 patients (Fig. 7A). Once more, *AKT3* behaved distinctly. Its expression level is highly correlated to a poor outcome (*p* = 0.0004), while *AKT2* expression cannot distinguish two groups with a significant difference (*p* = 0.4550). The data obtained with *AKT1* were very surprising. As opposed to *AKT3*, its expression level correlates significantly to a better survival (*p* = 0.0422).

Given these strong, yet opposite, predictive values of *AKT1* and *AKT3* expression levels, it was important to verify whether similar counterintuitive observations could be made in another consistent cohort. We therefore looked at the Leucegene public dataset encompassing 373 AML samples. As no clinical information can be obtained with the Leucegene cohort, were here downloaded the Kaplan–Meier overall survival curves as they appear online. We could not select the samples to provide information on a homogeneously treated set of patients. Yet, the results were very similar to those obtained with the Beat-AML cohort. High *AKT3* expression appears as a powerful predictor of poor survival (*p* = 0.0000), while in contrast *AKT1* shows as a strong predictor of better survival (*p* = 0.0012), and no significant difference can be highlighted using *AKT2* expression as a parameter (*p* = 0.1648) (Fig. 7B).

In each dataset, samples are split into 2 categories (AKT^high^ or AKT^low^) based on median gene expression value.

## Discussion

*Prognostic values of AKT1 and AKT3* That a higher expression of *AKT3* appears as a marker of bad prognosis is not surprising for a kinase lying in the PI3K-AKT-mTOR pathway so important for cancer cell growth and survival. What is quite counterintuitive, is the exact opposite prognostic value of *AKT1* expression. Our search in the literature of which of the AKT1 *versus* AKT3 kinase specific substrate(s) could provide a probable explanation remained unsuccessful. The expanding list of such specific substrates will certainly help elucidating this mystery. Yet, this discrepancy highlights the need to search for specific inhibitors capable of discriminating each of the three AKT protein isoforms. Up to now, the vast majority of experimental works designed at describing the function of AKT in cancer cells was performed using panAKT inhibitors. If the three AKT kinases have distinct or even opposite effects on a biological function, as is the case for instance in vascular tumors where AKT1 and AKT3 kinases exhibit opposite effects on endothelial cell growth^13^, this may have generated biases in data interpretation.

*What could be the advantage for HSCs or leukemic blasts to express AKT3?* As said in the introduction, a recent report indicates that unlike AKT1 and AKT2, the AKT3 kinase locates at the nuclear envelope, facing the cytosol, and its protein serine/threonine kinase activity appears constitutively activated in a panel of established cancer cells^6^. As no leukemic cell lines were included in this earlier study, it would be interesting to check whether leukemic blasts express also a constitutive AKT3 kinase. Given the cellular functions of AKTs this would confer a serious survival/proliferation advantage, notably in mature/monocytic-like blasts expressing a higher than expected level of AKT3 expression.

*Relationship between AKT3 and NPM1wt or NPM1c* A recent report showed that NPM1wt and NPM1c proteins exhibit opposite effects on AKT kinase activation in leukemic blasts. Indeed, it was shown there that the NPM1c protein physically associates with AKT and antagonizes the inhibitory effect of NPM1wt on AKT phosphorylation. Interestingly, this leads to enhanced sensitivity of *NPM1c* leukemic blasts to AKT inhibitors^14^. However, in this report were used only anti-AKT and anti-phosphoAKT antibodies that cannot discriminate the three AKT protein isoforms (i.e. panAKT antibodies). Therefore, it is yet not known whether NPM1c interacts preferentially with one or another of the AKT kinases. This merits further investigations.

*Relationship between AKT3 and RUNX1wt, RUNX1mut or RUNX1-RUNX1T1* Another striking observation in our study is the inverse correlations between elevated *AKT3* expression and *RUNX1mut* in one hand, and diminished *AKT3* expression and *RUNX1-RUNX1T1* translocation in the other hand, as compared to *RUNX1wt* samples. Here again, whether and how molecularly the two mutated or translocated *RUNX1* variants could control *AKT3* transcript expression in opposite ways, or whether these events are solely correlated with no causal connection, remains unknown. This warrants further investigation to experimentally determine whether in the myeloid lineage the RUNX1 transcription factor or its mutant or translocated variants interact directly with the *AKT3* gene locus. A recent report suggests that the RUNX1 transcription factor does indeed interact directly with the *AKT3* locus, although this was shown in lung^15^. In a clinical perspective, the very low expression of *AKT3* in *RUNX1-RUNX1T1* AML suggests it would be preferable to target AKT1 or AKT2 kinase in this leukemic entity. This is consistent with an earlier report which indicated that *RUNX1-RUNX1T1* blasts harbor an activated AKT1 kinase and are specifically sensitive to AKT1 inhibition both in vitro and in vivo settings^16^.

*Relationship between AKT3 and mutations related to MDS or MPN hematological disorders* The MDS- or MPN-related mutations affect genes involved in different molecular aspects of gene expression including RNA splicing (*SRSF2*, *U2AF1* and *SF3B1*), chromatin remodeling (*ASXL1*) and gene transcription (*BCOR*). It is therefore hard to anticipate how mutations in these different sorts of genes could impact *AKT3* expression. A quick glance at how *AKT3* RNA-seq reads align to the *AKT3* locus failed to reveal any change in the pattern of *AKT3* mRNA splicing that might have been responsible for the higher *AKT3* mRNA expression in splicing mutant samples (data not shown). In addition, as opposed to what was seen with *NPM1* or *RUNX1* genetic alterations, no correlation could be established between expression of *ASXL1* or *BCOR* (either wild types or mutants) to that of *AKT3* (data not shown), suggesting no direct link between the transcriptional activity of *ASXL1* or *BCOR* and the expression of *AKT3*.

*AKT3, genetic alterations and the degrees of blast maturation and aggressiveness* Taken together these data indicate that *AKT3* expression is highly variable among AML samples and that different kind of gene mutations/translocations are associated with either high or low *AKT3* expression. One feature which is more globally and positively correlated to *AKT3* expression is the degree of blast maturation (as opposed to healthy myeloid differentiation). We therefore favor a model whereby the causes of variable *AKT3* expression in AML may be multiple, but ultimately the most aggressive leukemic blasts are characterized by a high level of *AKT3*.

## Methods

### Donors, patients and tumor samples

Normal hematopoiesis. For studying normal hematopoiesis, three different datasets of the BloodSpot repository available on-line (https://www.bloodspot.eu)^9^ were used: (i) the dataset in the “*Normal human hematopoiesis (HemaExplorer)*” tab (here called bloodSpot #1); the dataset in the “Normal *hematopoiesis with AMLs”* tab (here called BloodSpot #2); (iii) the dataset in the “*Normal human hematopoiesis (DMAP)*” tab (here called BloodSpot #3). They all contain normalized and quantified micro-array data (log2) from normal hematopoiesis samples. See the figure legends for details on the micro-array probes. Were also used data from the Leucegene project (GSE48846 and GSE51954) containing poly(A) RNA-seq of sorted CD34+ HSC/progenitor cells from either cord of peripheral blood and sorted granulocytes or monocytes from peripheral blood.

IUCT-AML cohort. AML patients were diagnosed, sampled and treated in our medical center and registered at the HIMIP (Hémopathies INSERM Midi-Pyrénées, France) collection. In this study, 40 cytogenetically normal AML (CN-AML) samples collected at diagnosis and for which the genetic status is known (including mutations and chromosomal translocations) were analyzed. Detailed clinical data related to these 40 patients have been published earlier^9^.

Ethics approval. In accordance with French law, each patient was informed and the HIMIP collection has been declared to the Ministry of Higher Education and Research (DC 2008–307) and a transfer agreement has been obtained (AC 2008–129) after approbation by the local ethical committee (“Comité de Protection des Personnes Sud-Ouest et Outremer II”). Clinical and biological annotations have also been declared to the CNIL (“Comité National Informatique et Libertés”). This study was conducted in accordance with the Declaration of Helsinki.

### Beat-AML cohort

As a validation cohort for gene expression, were used the data of the Beat-AML project available on-line at http://vizome.org/aml2. This repository contains the genetic status (including mutations and chromosomal translocations) and quantified and normalized poly-(A) RNA-seq data of bone marrow or peripheral blood samples from healthy donors, and MDS, MPN and AML patients. In addition to sampling at diagnosis, a number of patient have also been collected at different times post-diagnosis. To explore a homogenous set of samples, the analyses in this study were performed only on AML samples that were collected at diagnosis and for which the genetic status and RNA-seq data are available, i.e. n = 445 AML samples.

### Overall survival Kaplan–Meier curves

For calculating the probability of survival, data from the Beat-AML and from the Leucegene projects were used. The Beat-AML cohort (http://vizome.org/aml2) proposes for each patient quantified and normalized poly-(A) RNA-seq (expressed in log2 RPKM) and matched survival data. For Kaplan–Meier curves the cohort was split into 2 categories based on median expression value of each AKT gene (see the results section for precisions on the samples that were included in survival analyses). The Leucegene projects (https://data.leucegene.iric.ca/survival?lang=en) proposes directly the relationship between gene expression and clinical outcome in terms of survival. The cohort is also split into 2 categories based on median gene expression value. The survival cohort consists of 373 diagnostic AML samples (excluding acute promyelocytic leukemia). Gene expression was generated using STAR/RSEM and GRCh38 (Gencode32) and is expressed in TPM.

For each cohort, overall survival (OS) time was calculated from the date of diagnosis until death or last received news. Surviving patients were censored at the date of last follow-up.

### RNA extraction, ribo-depletion and reverse transcription.

Total RNAs from the 40 CN-AML samples were extracted with the TRIzol reagent (Ambion, Austin, TX, USA). RNA integrity was evaluated using the RNA 6000 Nano Chip kit (Agilent Technologies, Massy, France). Only RNA extracts with RNA integrity values ≥ 7 underwent further reverse transcription. rRNA depletion was performed from total RNA with Ribo-Zero™ rRNA Removal Kits (Epicentre, Madison, WI, USA). All samples were reverse transcribed using the Superscript II reverse transcription kit (Invitrogen, Beijing, China), according to the manufacturer's protocol.

### RNA-seq

RNA sequencing of the 40 CN-AML samples was performed at the BGI (Hong Kong). Paired-end, strand-specific reads of ~ 100 nucleotides were generated on an Illumina HiSeqTM2000. Alignment and mapping were performed using Tophat1 against the hg19 genome and the mapped reads were assembled by Cufflinks 2.0.22. The Cuffcompare program was used to merge the RefSeq, ENCODE and UCSC human known genes freeze January 2013 into one gene annotation set for comparison with the assembled transcripts^17^.

### Statistical analyses

Unpaired Student t test (*: *p* < 0.05, **: *p* < 0.01, ***: *p* < 0.005, ****: *p* < 0.001) was used for sample inter-group significance. Linear regression (r^2^) was used for trend curves. Log-rank test was used for Kaplan–Meier curves.

## Data Availability

The raw and processed RNA-sequencing data generated in this study have been deposited at the National Center for Biotechnology Information Gene Expression Omnibus (repository number GSE62852).
